# Transmission of SARS-CoV-2 in Public Transportation Vehicles: A Case Study in Hunan Province, China

**DOI:** 10.1093/ofid/ofaa430

**Published:** 2020-09-13

**Authors:** Kaiwei Luo, Zhao Lei, Zheng Hai, Shanliang Xiao, Jia Rui, Hao Yang, Xinping Jing, Hui Wang, Zhengshen Xie, Ping Luo, Wanying Li, Qiao Li, Huilu Tan, Zicheng Xu, Yang Yang, Shixiong Hu, Tianmu Chen

**Affiliations:** 1 Hunan Provincial Center for Disease Control and Prevention (Workstation for Emerging Infectious Disease Control and Prevention, Chinese Academy of Medical Sciences), Changsha City, Hunan Province, People’s Republic of China; 2 State Key Laboratory of Molecular Vaccinology and Molecular Diagnostics, School of Public Health, Xiamen University, Xiamen City, Fujian Province, People’s Republic of China; 3 Shaodong Municipal Center for Disease Control and Prevention, Shaodong City, Hunan Province, People’s Republic of China; 4 Shaoyang Municipal Center for Disease Control and Prevention, Shaodong City, Hunan Province, People’s Republic of China; 5 Department of Biostatistics, College of Public Health and Health Professions, Emerging Pathogens Institute, University of Florida, Gainesville, Florida, USA

**Keywords:** SARS-CoV-2, COVID-19, probable airborne transmission, vehicles

## Abstract

Here we report a case study of a severe acute respiratory syndrome coronavirus 2 (SARS-CoV-2) outbreak event during bus trips of an index patient in Hunan Province, China. This retrospective investigation suggests potential airborne transmission of SARS-CoV-2 and the possibility of superspreading events in certain close contact and closed space settings, which should be taken into account when control strategies are planned.

The pandemic of coronavirus disease 2019 (COVID-19) is imposing a serious threat to global public health and the economy. While the World Health Organization (WHO) and the US Centers for Disease Control and Prevention (CDC) have stated that direct contact with respiratory droplets from sneezing and coughing patients or contaminated fomites is the predominant transmission route of severe acute respiratory syndrome coronavirus 2 (SARS-CoV-2), the causative pathogen for COVID-19, there have been speculations about the possibility of aerosol transmission and its contribution to the pandemic [1, 2]. Aerosols containing viable SARS-CoV-2 particles have been detected in hospitals [3, 4] and have been shown to last for 3 hours in laboratory conditions [5]. In addition, there have been several studies reporting transmission events from asymptomatically infected individuals or from COVID-19 patients during their presymptomatic incubation period, further indicating the possibility of aerosol transmission [6, 7]. Here we report a contact-tracing study on a COVID-19 outbreak event involving public transportation in Hunan Province, China. In this outbreak, we identified 10 lab-confirmed infections directly associated with exposure to a single COVID-19 patient during bus trips. This case study adds useful information to our understanding of the transmission route of SARS-CoV-2.

The primary case (Patient A), who worked at Place I, had symptom onset on January 22 and tested positive for SARS-CoV-2 on January 29. According to a retrospective investigation, 5 and 3 days before his onset of symptoms, respectively, he had meals and work-related contact with his colleague (infection source) who had onset on January 14 and tested positive on January 16, 2020. On January 22, 2020, Patient A traveled without wearing a face mask from Place I to Place III via public transportation, with a transfer at Place II. The first ride on a tour coach took 2.5 hours, and the second ride on a minibus took about 1 hour. After he was confirmed to have COVID-19, a total of 243 close contacts of Patient A and subsequently identified infections were traced and monitored.

## METHODS

According to the guidelines of the New Coronavirus Pneumonia Prevention and Control Program (4th edition) published by the National Health Commission of China [8], nasopharyngeal swabs were collected from suspected cases, including Patient A and contacts with subsequent illness onsets and all traced close contacts of confirmed cases. The specimens underwent a real-time reverse transcription polymerase chain reaction (RT-PCR) assay [9]. In addition, an epidemiological survey was administered on each suspected case to inquire about his or her travel history and close contacts. Details regarding the seating arrangement on the buses and loading and unloading stops of all passengers were obtained from the public transportation authority.

## RESULTS

**Figure 1.**
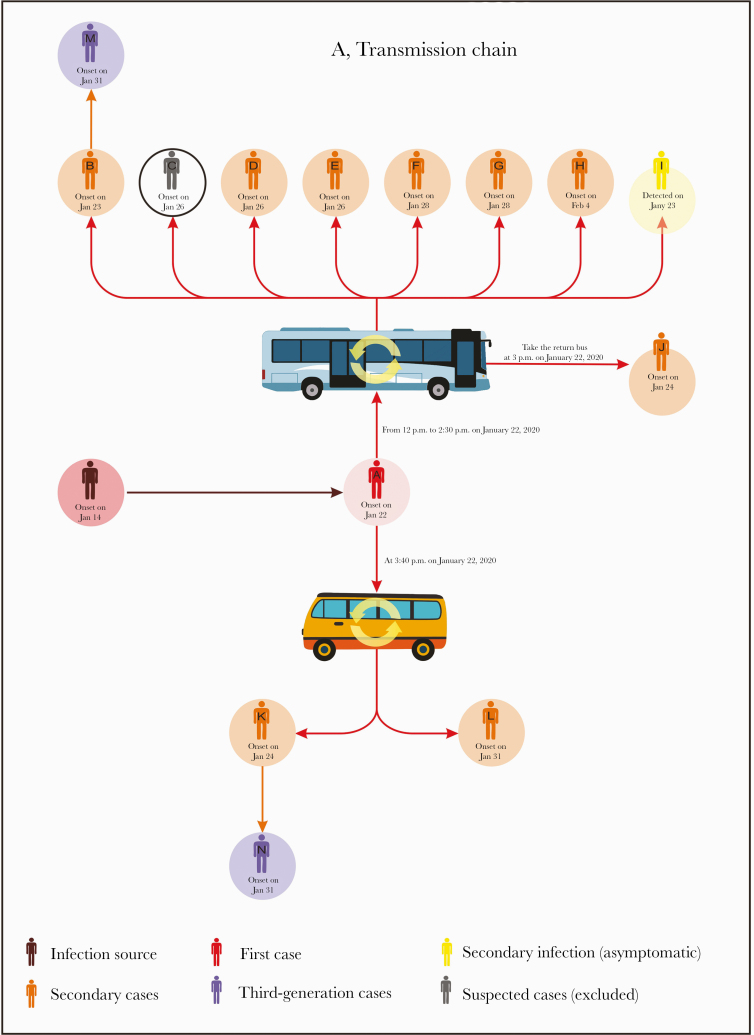

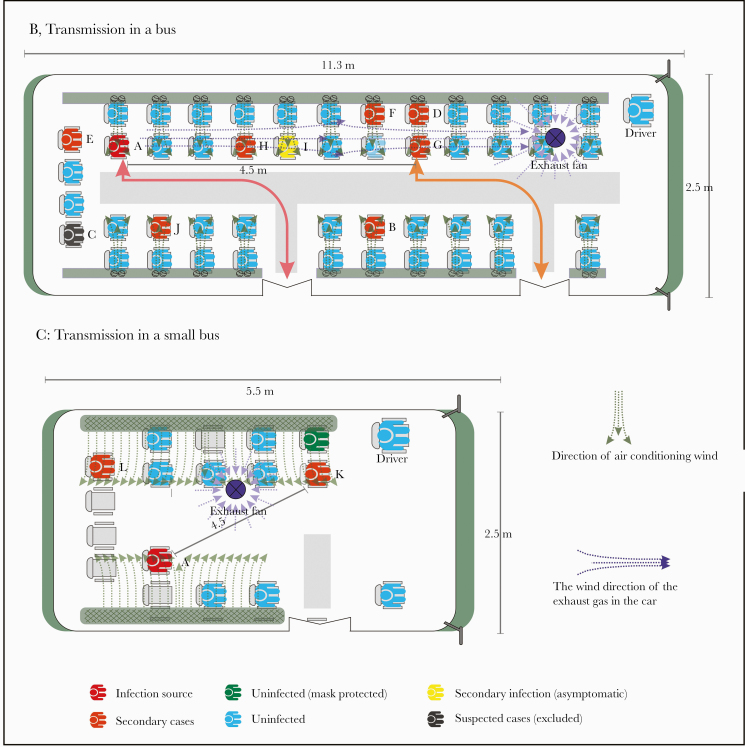
The transmission chain of severe acute respiratory syndrome coronavirus 2 and the seat arrangement of passengers in public transportation vehicles. A, The transmission chain of a coronavirus disease 2019 outbreak on buses in Hunan Province, China. Arrows indicate transmission links and directions. B, The seat arrangement of passengers who shared a ride with the index patient on a tour coach. Passengers are colored by disease status: index case (red), secondary cases (orange), secondary asymptomatic infection (yellow), and uninfected passengers (blue). Arrows indicate the paths of boarding and debussing. C, The seat arrangement of passengers who shared a ride with the index patient on a minibus. Passengers are colored by disease and face mask–wearing status: index case (red), secondary cases (orange), and uninfected passengers (green if wearing a mask and blue if not).

A total of 243 individuals, in addition to Patient A, were investigated for the cluster of COVID-19 cases who were epidemiologically linked to the bus trips of Patient A on January 22, 2020. Of these individuals, 12 tested positive and were confirmed to be COVID-19 cases. The remaining 231 test-negative individuals returned home after a 14-day quarantine period. The detailed timeline of illness onset for all cases is presented in Figure 1A.

The tour coach was 11.3 m long and 2.5 m wide with 49 seats, fully occupied with all windows closed and the ventilation system on during the 2.5-hour trip. Among the 49 passengers (including the driver) who shared the ride with Patient A (Figure 1B), 8 tested positive and 8 developed symptoms (Patient B had onset on January 23, Patients C, D, and E on January 26, Patients F and G on January 28, and Patient H on February 4), with a single asymptomatic infection (Patient I). Patient C was finally excluded because he/she tested negative for SARS-CoV-2 virus by RT-PCR 3 times on different days and negative for antibodies including IgM and IgG. Patient A sat at the second rear row, and the other 9 infected passengers were distributed over the middle and rear rows. The nearest infectee was Patient E (with onset 4 days later), who sat right behind Patient A, about 1 m away. The furthest infectees were Patient D and Patient G, who sat 7 rows (~4.5 m) away from Patient A and became ill 4 and 6 days later, respectively. The retrospective survey indicated that Patients A and G got on and off through different doors of the bus and had no direct contact with each other during the trip. The ventilation system and possible directions of airflow in the bus are shown in Figure 1B.

After arrival at Place II, the tour coach parked for 30 minutes, without any disinfection, and then loaded another group of passengers and returned to Place I. Among the 49 passengers (excluding the driver) on the return trip, Patient J, who sat in close proximity to the seat that Patient A had occupied during the last trip, had symptom onset on January 24.

During the trip of Patient A from Place II to Place III on the minibus (Figure 1C), 2 (Patients K and L) out of 12 passengers (including the driver) were diagnosed with COVID-19, with symptom onset on January 24 and 31, respectively. The minibus is about 5.5 m long and 2.5 m wide with 18 seats. All windows were closed during the 1-hour trip. Patient A was seated 1 row (about 1.5 m) away from Patient L and 3 rows (~4.5 m) away from Patient K. The ventilation system and possible airflow directions in the minibus are shown in Figure 1C.

**Figure 2.**
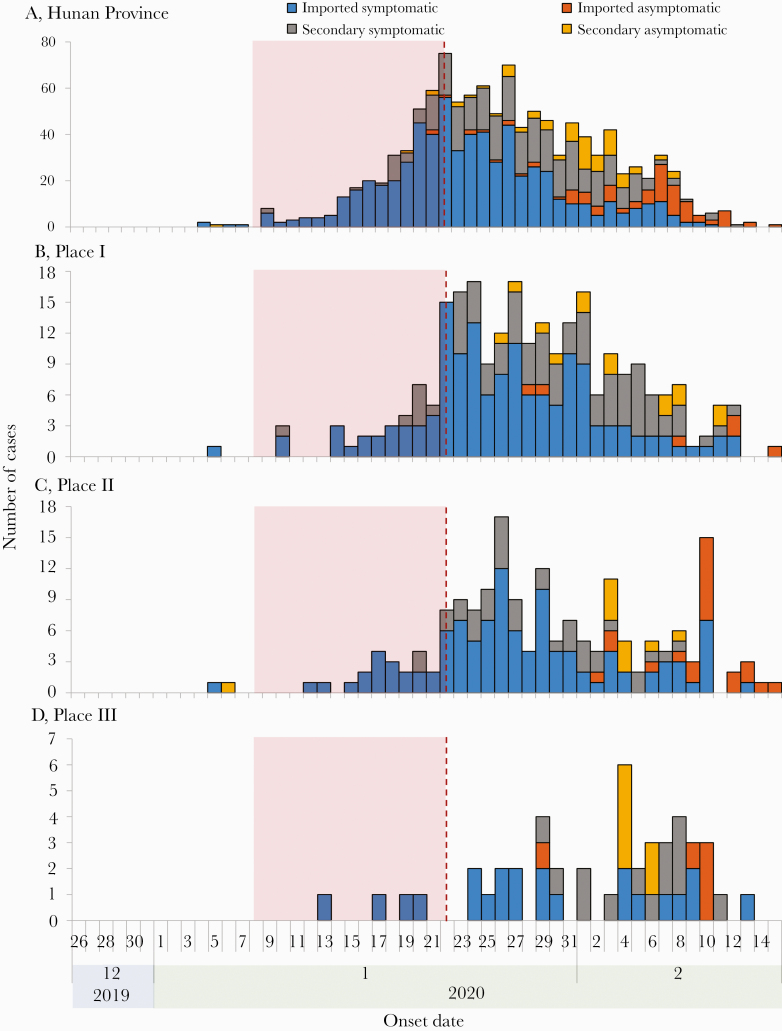
Epidemic curves of coronavirus disease 2019 (COVID-19) in Hunan Province and its 3 subregions related to this study. Cases were classified as imported symptomatic cases, imported asymptomatic cases, locally infected symptomatic cases, and locally infected asymptomatic cases. Epidemic curves of COVID-19 are shown for (A) Hunan Province; (B) the city where Place I is located; (C) the city where Place II is located; and (D) the county where Place III is located.

None of these 10 secondary cases wore face masks during the rides. We found 2 tertiary cases, Patients M and N, who are cousins of the secondary cases Patients B and K, respectively. Patients M and N had been living with their cousins since January 22 and developed symptoms on January 31. None of these 12 secondary and tertiary cases had traveled to Wuhan City, the COVID-19 epicenter of China, or had been exposed to other COVID-19 patients in the 2 weeks before their symptom onset. The provincial surveillance data indicate that there were few locally infected cases in Hunan Province before January 22, 2020; in particular, only 7, 2, and 0 locally infected cases were reported before January 22 in the cities or counties where Places I, II, and III are located, respectively (Figure 2). According to epidemiological surveys on all secondary and tertiary cases, none of these individuals had travel history to Wuhan or nearby cities, and no known infections were reported in their workplaces or communities during the 14 days before January 22, 2020. The median incubation period among the secondary cases (range) was 4 (1–13) days. Excluding Patient J and focusing only on the 2 bus trips of Patient A, we estimated the secondary attack rate (SAR) during an exposure period of up to 2.5 hours on a bus to be 9/(48 + 12) = 15.00% (95% CI, 6.00%–24.00%).

## DISCUSSION

This outbreak on public transportation vehicles highlighted the efficient transmission of SARS-CoV-2 in crowded and closed settings. According to the WHO, the main transmission routes of COVID-19 appear to be respiratory droplets or direct contact with fomites [8]. Transmission via droplets is usually confined to distances within 2 m; however, the majority of secondary cases were seated more than 2 m away from Patient A. Some cases might have been infected by touching fomites. On the other hand, this transmission route is unlikely to explain every secondary case; for example, Patient G was never physically close to Patient A or where he walked during the whole trip. The closed windows with running ventilation on the buses could have created an ideal environment for aerosol transmission [10]. Aerosol transmission occurs when microorganisms are contained in droplet nuclei of a size <5–10 μm, which can remain suspended in the air and thus travel relatively far [11, 12]. On the tour coach, the ventilation inlets were aligned above the windows on both sides, and the exhaust fan was in the front, possibly creating an airflow carrying aerosols containing the viral particles from the rear to the middle and front of the vehicle. Consequently, aerosol transmission cannot be ruled out.

Several limitations are inherent in our study: (1) we could not verify transmission via fomites as no environmental samples were collected; (2) the SAR was likely overestimated as it is solely based on a single large cluster; (3) there might be recall bias because the information (including the seat number) was collected retrospectively; (4) no viral genetic sequence data were available from these cases to prove linkage; and (5) some of the secondary and tertiary cases could have been exposed to unknown infections, especially asymptomatic ones, before or after the bus trips. Given the potential for fomites and aerosol transmission of SARS-CoV-2, we recommend timely disinfection of public transportation vehicles and an “open window” policy whenever possible. It is also crucial for all individuals, regardless of respiratory symptoms, to wear face masks and to maintain hand hygiene when they use public transportation.
